# Fetal-Type Rhabdomyoma of the Cheek: A Conservative Management

**DOI:** 10.3390/children10111818

**Published:** 2023-11-16

**Authors:** Angela Troisi, Valentina Pelliccia, Bruna Malta, Vincenzo Domenichelli, Federico Marchetti

**Affiliations:** 1 Department of Pediatrics, Santa Maria delle Croci Hospital, 48121 Ravenna, Italy; angela.troisi@auslromagna.it (A.T.); valentina.pelliccia@auslromagna.it (V.P.); 2 Department of Radiodiagnostics, Santa Maria delle Croci Hospital, 48121 Ravenna, Italy; bruna.malta@auslromagna.it; 3 Pediatric Surgery, Infermi Hospital, AUSL della Romagna, 47923 Rimini, Italy; vincenzo.domenichelli@auslromagna.it

**Keywords:** tumor, infant, head, rhabdomyoma, child, conservative management, case report

## Abstract

Extracardiac rhabdomyomas are rare benign mesenchymal tumors diagnosed upon radiological and hystologic investigations and the treatment of choice is surgical exertion. There aren’t any similar cases managed conservatively reported in literature as in our case, to the best of our knowledge. We present a rare case of fetal cheek rhabdomyoma diagnosed in a healthy 2 months-old boy, with asymptomatic mass over the left masseter. The lesion could not be removed, due to the size and dimensions and the young age of the patient. However, the lesion did not show signs of spreading or progression over a 36 months follow-up. Fetal rhabdomyoma is a benign tumor, often located in the head and neck district, where surgery, especially in very young children, is associated with a high risk of complications and long-term sequelae. Our case report demonstrates the possibility to manage these lesions conservatively in the first years of life with close sonographic and clinical follow-up.

## 1. Introduction

Rhabdomyomas are rare benign tumors arising from the striated muscle and cardiac rhabdomyomas account for the most common type. The extracardiac rhabdomyomas account for less than 2% of all neoplasm showing striated muscle differentiation and 70 to 90% of all extracardiac rhabdomyomas occur in the head and neck area [1,2]. Those are further classified into adult type, genital type, and fetal type. The fetal type is the least common and generally occurs in male infants less than 3 years of age [1]. Fetal rhabdomyomas have a benign biological behavior showing myotube-like differentiation, therefore resembling primitive skeletal muscle fibers during fetal development. They grow slowly, with little or no change in size or histologic features over time; therefore, complete excision is usually curative and recurrence after surgical removal is extremely rare [3]. Despite their rarity, fetal rhabdomyomas should always be of concern for pathologists, as they may often resemble embryonal rhabdomyosarcoma [2,3]. We present a rare case of fetal cheek rhabdomyoma diagnosed in a 2 months-old boy who did not receive surgical excision of the lesion. The boy was instead closely monitored over a 36 months follow-up and the tumor did not show any signs of progression at 12, 24, and 36 months sonographic and clinical review.

## 2. Case Presentation

**Figure 1:**
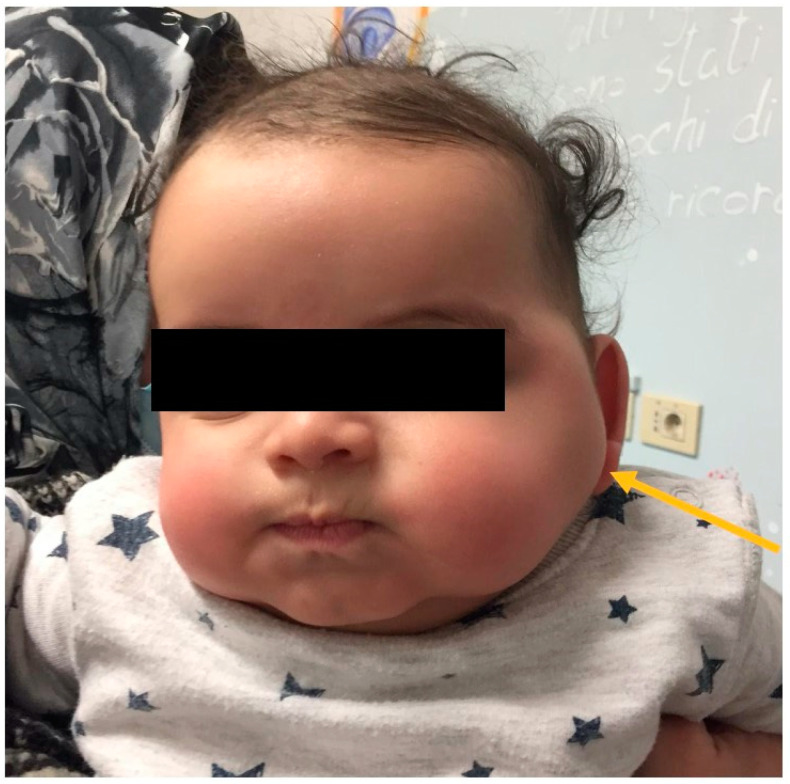
Swelling of the left cheek in a 2 months-old boy.

A healthy 2 months-old boy, without significant family history, presented to our clinic with a newly noted mass over the left cheek over the masseter area (Figure 1).

**Figure 2:**
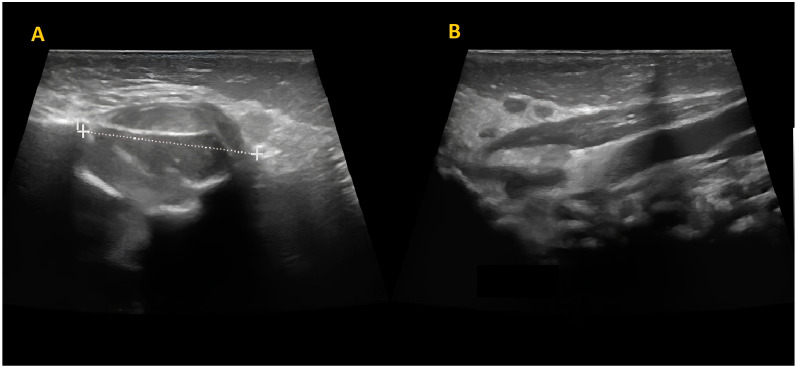
Skin and subcutaneous ultrasound. A fusiform mass over the left masseter muscle, inhomogeneous and hypoechogenic, sized 17 mm × 15 mm (**A**). Concurrent thickening of the left sternocleidomastoid muscle at the mastoid head with increased thickness compared to the contralateral side (8 mm) (**B**).

The mass was firm, partially mobile, cold, and apparently painless, with a diameter of around 2 cm. The lesion did not impair neck rotation and lateral movements, neither caused forced head position or tilting. An ultrasound study was performed and showed a fusiform mass over the left masseter muscle, which appeared inhomogeneous and hypoechogenic, sized 17 mm × 15 mm. The mastoid extremity of the homolateral sternocleidomastoid muscle (SCM) was also noted to be thickened (8 mm) (Figure 2).

**Figure 3:**
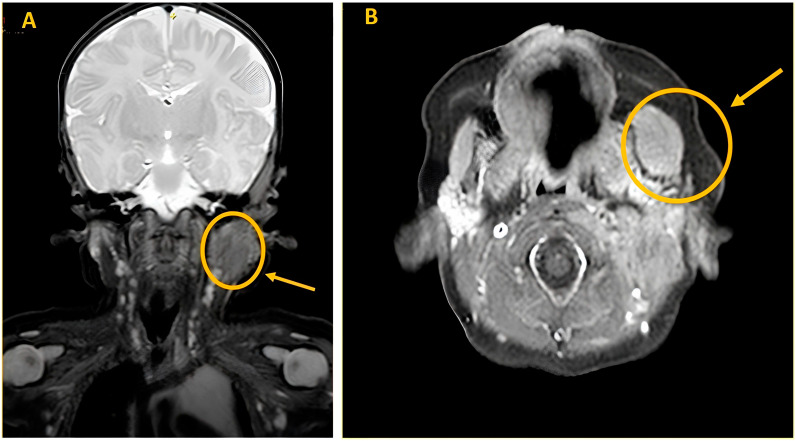
Head and neck MRI. Examination performed before and after intravenous bolus administration of 1 ml of paramagnetic contrast medium, such as Dotarem (gadoteric acid) 0.5 mmol/mL. (**A**) Coronal image T2 dixon: Fusiform dilation of the left masseter muscle (20 mm × 15 mm × 21 mm) with minimal, homogeneous hyperintensity in T2 signal. Fusiform swelling at the level of the cranial slope of the left sternocleidomastoid muscle (13 mm × 14 mm × 31 mm). (**B**) Intravenous contrast-enhanced axial image T1 dixon: Homogeneous isointensity of the signal in T1 with no perifocal edema or pathological enhancements after contrast administration.

A Magnetic Resonance imaging (MRI) of the soft tissues was performed and showed an enlargement in the context of the left masseter muscle (20 mm × 15 mm × 21 mm). The lesion appeared minimally hyperintense in T2 sequences and isointense in T1 sequences with negative contrast enhancement. The MRI study confirmed a thickened proximal SCM (13 mm × 14 mm × 31 mm), as shown in Figure 3.

Further ultrasound studies were performed in the following months and showed rapid mass growth (around 10 mm over 2 months). Hence, in view of the growth rate and in order to confirm the nature of the lesion, a punch biopsy was performed. The pathology report showed morphological and immunohistochemical characteristics of fetal rhabdomyoma, intermediate type. Microscopic description of histological examination was: Proliferation predominantly cellular, composed partly of fused cells with abundant eosinophilic cytoplasm, and partly of round cells with a decentered nucleus and intensely eosinophilic, roundish cytoplasm, suggestive of rhabdomyoblasts. Small oval or fused nuclei without atypia. No evidence of necrosis or pleomorphism. No evidence of mitosis. On immunohistochemical examination, the neoplastic population was diffusely positive for Myogenin, Myo-d1, desmin, muscle-specific Actin, and negative for smooth muscle Actin, myosin, CD34. Proliferation fraction assessed with Ki67 antibody was 2%.

**Figure 4:**
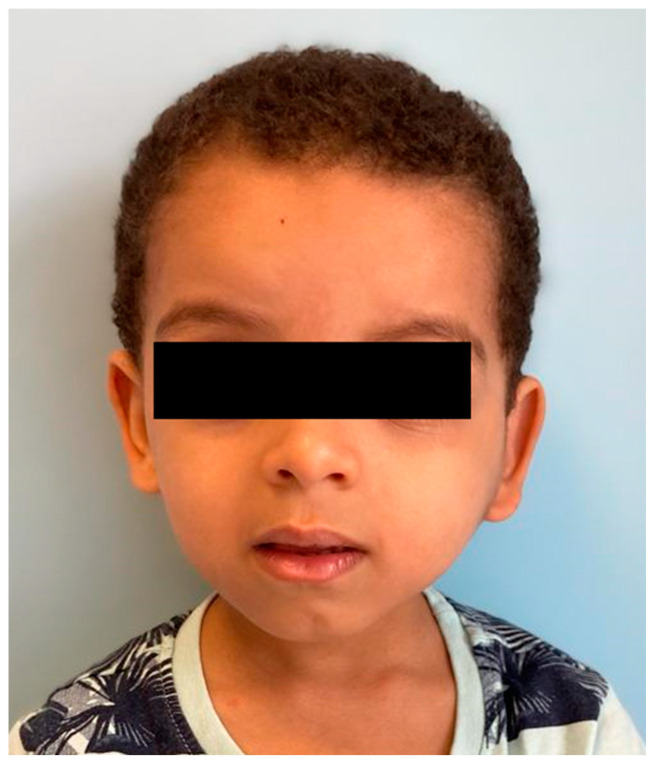
Picture of the child at 36 months follow-up, unchanged tumor size.

A total excision of the tumor was deemed not feasible, because of the size and location of the mass alongside the young age of the patient. Conversely, it was discussed and agreed with the boy’s family a close follow-up, with period clinical and ultrasound sonographic (USS) assessments. The ultrasounds performed at 12, 24, and 36 months showed an unchanged tumor size without any signs of spreading or progression despite the kid’s growth (Figure 4).

## 3. Discussion

Rhabdomyomas are rare benign mesenchymal tumors with skeletal muscle differentiation. Unlike most other soft tissue tumors, rhabdomyomas are strikingly outnumbered by their malignant counterpart (rhabdomyosarcomas), and they account only for 2% of all tumors with striated muscle differentiation [2,3]. They are divided in two groups based on their localization: cardiac rhabdomyomas, that occur almost exclusively in infants with Tuberous Sclerosis, and extracardiac rhabdomyomas. These latter are fairly more uncommon with only 150 cases published by case reports, and further divided into three clinical and morphological different subtypes: adult type, which commonly affects elder people; genital type, usually affecting middle-aged women [3]; and the fetal type. The latter is the least common of all rhabdomyomas and affects mostly male infants aged less than 3 years, and it usually evolves and grows until this age. About 25% of fetal rhabdomyoma are congenital [1,4].

Both adult and fetal rhabdomyoma affect mostly the head and neck area (90% of cases), such as the parapharyngeal space, salivary glands, larynx, mouth, and neck soft tissue. Uncommon locations for adult and fetal rhabdomyoma include the mediastinum or the chest for the former and abdomen and extremities for the latter. Both types usually have a slow growth rate, but some may present a faster growth pace, causing symptoms such as hoarseness, airway obstruction, dysphagia, and decreased vision. 

The myxoid type of fetal rhabdomyomas (classic type) is composed of spindle-shaped or primitive oval immature skeletal muscle fibers with abundant myxoid backgrounds. Conversely, the juvenile (intermediate) type shows less myxoid matrix and various differentiated skeletal muscle fibers, while the cellular type is characterized by uniform populations of differentiating myoblasts [5].

Three different types of extracardiac rhabdomyoma (adult, fetal and genital type) can be distinguished by using light microscopy and immunohistochemical markers (fetal rhabdomyoma more often expresses desmin, muscle specific actin and myoglobin, and are negative for myogenin and S-100 protein [6,7]). Differential diagnosis includes neoplastic and non neoplastic condition. Amongst the former rhabdomyosarcoma is the most common, but also granular cell tumor (GCT), hibernoma, reticulohistiocytoma and lymphoma can present in a similar fashion and need to be ruled out. Amongst the and non-neoplastic conditions the main differential diagnosis are represented by fibromatosis colli, nodular fasciitis, proliferative myositis, that can mimic muscle tumors [4]. Imaging studies like ultrasonography and MRI help during the initial differential diagnostic process, however the final diagnosis is determined by histological and immunohistochemical tests [5,7]. The embryonal and spindle cell type of rhabdomyosarcomas are the main histological differential diagnosis. In fact, fetal rhabdomyomas are usually located superficially (dermis and subcutaneous tissue) and show pushing margins, conversely rhabdomyosarcomas have infiltrative margins and are found in deeper location (intramuscular layers). Moreover, marked cellular atypia and pleomorphism, atypical mitosis and necrotic areas are never found in fetal rhabdomyomas, whereas mild cellularity, pleomorphism and mitotic activity are features that may be encountered. Therefore, when the former set of features are described a diagnosis of rhabdomyosarcoma should be considered [3]. Additionally, also GCT should be considered and ruled out since this tumor can undergo malignant transformation. Immunohistochemically GCT and hibernoma can be identified as they are both positive for S-100 protein, but skeletal muscle differentiation is not present [5,7].

Since their benign biological behavior (malignant transformation has not been described [3,7]), the recommended approach for rhabdomyoma is complete surgical excision of the lesion with rare recurrences being reported (16% of cases) following a complete removal [1,3]. Few recurrent cases have been described but they were likely attributable to incomplete resection [6].

The peculiar aspect of our case report is represented by the successful conservative management of a rare tumor, which, to the best of our knowledge, has never been treated without a surgical approach. Also, the peculiarity of the case is represented by a rare diagnosis for a common children issue such as facial soft tissue swelling.

Head and neck district surgery, especially in very young children, is associated with a high risk of complications. Surgery to remove a lesion in the masseter muscle could potentially result in injuries to the muscle itself, the parotid gland, as well as some branches of both the trigeminal and facial nerves. This can lead to disruptions in the development of chewing, swallowing, and speech functions during a crucial growth phase. It may also cause paralysis of the facial expression muscles and alterations in the sensitivity of certain areas of the tongue and the lower part of the face, as well as functional changes in the salivary glands. Equally important to consider is the potentially destructive scarring outcome, with both aesthetic and functional consequences.

Our case report demonstrates the possibility to manage conservatively a rhabdomyoma of the cheek in the first years of life, when it is not associated with any other symptoms, provided a close clinical and radiological follow-up is feasible.

Obviously, the “wait and watch” approach can always be reconsidered in case of clinical changes, such as a significant increase in size of the lesion and/or the onset of compressive symptoms; in those situations, surgery should be rediscussed. However, if the mass remains stable in size and clinically silent over time, excision surgery can be considered after the first developing years of infancy to limit interferences with crucial skills development of this period, such as solid food swallowing or vocalizations and phonation finalized to speech production.

## Data Availability

Data are contained within the article.
